# Familial melanoma-astrocytoma syndrome: synchronous diffuse astrocytoma and pleomorphic xanthoastrocytoma in a patient with germline CDKN2A/B deletion and a significant family history 

**DOI:** 10.5414/NP301022

**Published:** 2017-07-12

**Authors:** Andrew K. Chan, Seunggu J. Han, Winward Choy, Daniah Beleford, Manish K. Aghi, Mitchel S. Berger, Joseph T. Shieh, Andrew W. Bollen, Arie Perry, Joanna J. Phillips, Nicholas Butowski, David A. Solomon

**Affiliations:** 1 Department of Neurological Surgery,; 2 Department of Pediatrics,; 3 Institute for Human Genetics,; 4 Division of Neuropathology, Department of Pathology, and; 5 Clinical Cancer Genomics Laboratory, Department of Pathology, University of California, San Francisco, CA, USA

**Keywords:** glioma predisposition syndrome, CDKN2A, p16INK4a, diffuse astrocytoma, pleomorphic xanthoastrocytoma, nerve sheath tumor, melanocytic nevi

## Abstract

Familial melanoma-astrocytoma syndrome is a tumor predisposition syndrome caused by inactivating germline alteration of the *CDKN2A* tumor suppressor gene on chromosome 9p21. While some families with germline *CDKN2A* mutations are prone to development of just melanomas, other families develop both melanomas, astrocytomas, and occasionally other nervous-system neoplasms including peripheral nerve sheath tumors and meningiomas. The histologic spectrum of the astrocytomas that arise as part of this syndrome is not well described, nor are the additional genetic alterations that drive these astrocytomas apart from the germline *CDKN2A* inactivation. Herein, we report the case of a young man with synchronous development of a pleomorphic xanthoastrocytoma, diffuse astrocytoma, and paraspinal mass radiographically consistent with a peripheral nerve sheath tumor. His paternal family history is significant for melanoma, glioblastoma, and oral squamous cell carcinoma. Genomic profiling revealed that he harbors a heterozygous deletion in the germline of chromosome 9p21.3 encompassing the *CDKN2A* and *CDKN2B* tumor suppressor genes. Both the pleomorphic xanthoastrocytoma and diffuse astrocytoma were found to have homozygous deletion of *CDKN2A/B* due to somatic loss of the other copy of chromosome 9p containing the remaining intact alleles. Additional somatic alterations included *BRAF* p.V600E mutation in the pleomorphic xanthoastrocytoma and *PTPN11*, *ATRX*, and *NF1* mutations in the diffuse astrocytoma. The presence of germline *CDKN2A/B* inactivation together with the presence of multiple anatomically, histologically, and genetically distinct astrocytic neoplasms, both with accompanying somatic loss of heterozygosity for the *CDKN2A/B* deletion, led to a diagnosis of familial melanoma-astrocytoma syndrome. This remarkable case illustrates the histologic and genetic diversity that astrocytomas arising as part of this rare glioma predisposition syndrome can demonstrate.

## Introduction 

Family history of brain tumors is an important risk factor in a small fraction of patients with primary glial neoplasms of the central nervous system [1, 2]. Among these patients, a subset harbor pathogenic germline alterations as part of well-characterized tumor predisposition syndromes [3]. These include germline mutations or deletions affecting the *NF1* tumor suppressor gene as part of neurofibromatosis type 1 syndrome, *TP53* tumor suppressor gene as part of Li-Fraumeni syndrome, and mismatch repair genes (e.g. *MLH1*, *MSH2*, *MSH6*, *PMS2*) as part of Lynch/Turcot syndrome. Multiple additional glioma susceptibility genes have been more recently identified. These include *POT1*, which encodes a protein involved in telomere maintenance, in which inactivating germline mutations have been identified in families with multiple members affected by oligodendroglioma [4]. Consortiums, such as GLIOGENE, are currently performing genome-wide association studies on large numbers of patients with primary glial neoplasms in order to determine the complete spectrum of genetic variants that increase risk of glioma development [5]. 

In addition to the aforementioned genetic syndromes, familial melanoma-astrocytoma syndrome has emerged as a rare cause of inherited glioma predisposition. Kaufman et al. [6] first described in 1993 a family in which cutaneous malignant melanoma or cerebral astrocytoma were seen in eight members over three generations and therefore suggested the presence of a possible new genetic syndrome. A study in 1995 examined the incidence of tumors of the nervous system as second cancers or in family members of 904 patients with cutaneous melanoma, which identified 15 families with cutaneous melanoma in which one or more family members had nervous system tumors including astrocytoma, glioblastoma, meningioma, and “acoustic neurilemmoma” (now referred to as vestibular schwannoma) [7]. Then a third study in 1997 described a family with a cancer syndrome including cutaneous melanoma, dysplastic nevi, astrocytoma, neurofibroma, schwannoma, and meningioma [8]. Together, these epidemiologic studies identified the presence of a distinct tumor predisposition syndrome that includes increased risk for both melanoma and nervous system tumors, predominantly astrocytomas. 

The genetic basis of this tumor predisposition syndrome was elucidated in 1998 by Bahuau et al. [9] who reported identification of deletions of the INK4 locus at chromosome 9p21.3 in both of the families described in the original 1993 and 1997 reports. The INK4 locus contains, in close proximity, both the *CDKN2A* and *CDKN2B* tumor suppressor genes. *CDKN2A* encodes the p16INK4a cyclin-dependent kinase inhibitor and in an alternative reading frame also encodes p14ARF, an inhibitor of p53 signaling, while *CDKN2B* encodes p15INK4b, a cyclin-dependent kinase inhibitor with a high degree of homology to p16INK4a. *CDKN2A* had already been recognized as one of the major susceptibility genes for familial cutaneous melanoma, most commonly due to inactivating point mutations but also occasionally gene deletions [10, 11]. Familial melanoma-astrocytoma syndrome is now appreciated to represent an autosomal-dominant variant of the familial melanoma syndrome caused by heterozygous germline *CDKN2A* inactivation that also includes development of astrocytomas and occasionally other neural tumors including peripheral nerve sheath tumors and meningioma (Online Mendelian Inheritance in Man, entry # 155755). 

Since the initial reports, a few additional families with genetically-confirmed familial melanoma-astrocytoma syndrome have been described [12, 13, 14, 15, 16, 17]. However, the histologic features of the astrocytomas that arise as part of this syndrome are not well described, nor are the somatic genetic alterations that drive these astrocytomas in addition to the germline *CDKN2A* inactivation. Herein, we report the case of a young man with a family history of melanoma, glioblastoma, and oral squamous cell carcinoma who was found to have synchronous development of a pleomorphic xanthoastrocytoma, diffuse astrocytoma, and a paraspinal mass radiographically consistent with a peripheral nerve sheath tumor. Pathologic and genomic assessment demonstrated the presence of germline *CDKN2A/B* deletion diagnostic of familial melanoma-astrocytoma syndrome, and also revealed the diversity of histologic features and genetic alterations that can be seen in astrocytomas arising as part of this rare glioma predisposition syndrome. 

## Case report 

**Figure 1.**
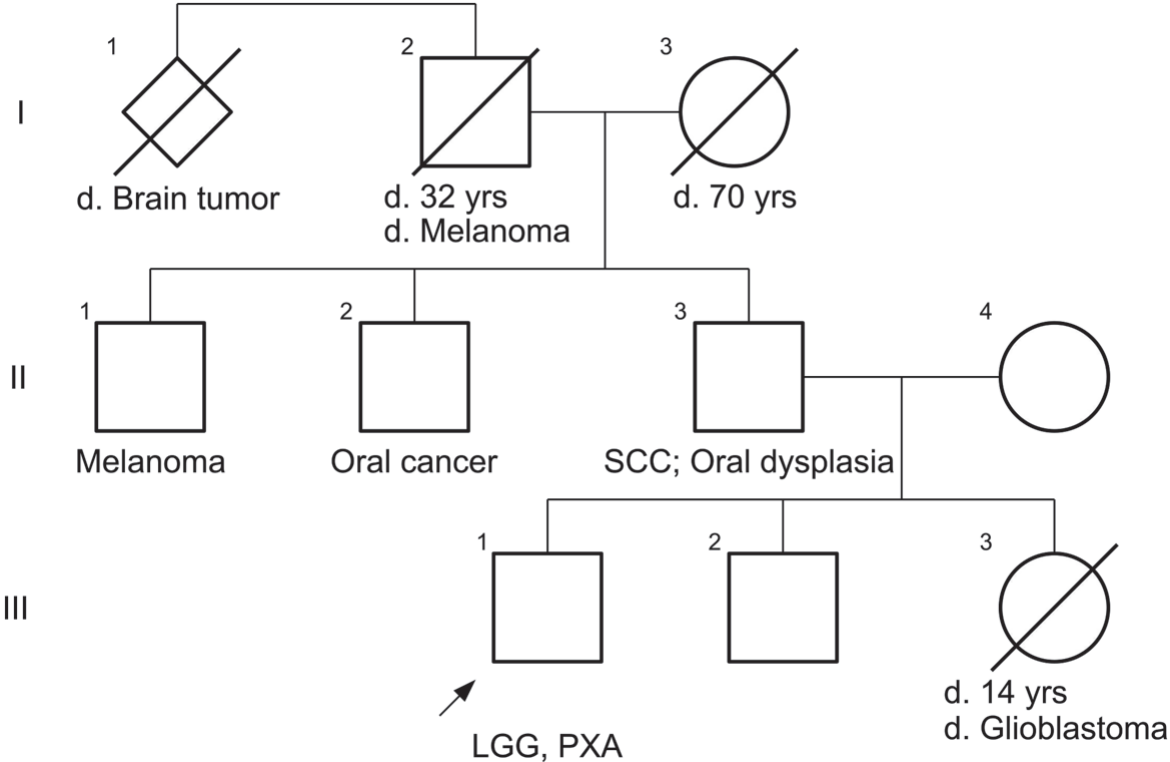
Pedigree of the patient’s family and paternal lineage. The proband is marked with an arrow. SCC = squamous cell carcinoma of the oropharynx; LGG = low-grade glioma (diffuse astrocytoma); PXA = pleomorphic xanthoastrocytoma.

A 23-year-old Caucasian man initially presented with new-onset generalized tonic-clonic seizures. He was found to have a non-enhancing lesion in the left frontal lobe and underwent resection which demonstrated a low-grade astrocytoma. Evaluation of *IDH1/2*, *ATRX*, *TP53*, and *BRAF* status was not performed, and this specimen is not currently available for our pathologic review and genetic analysis. Dermatologic evaluation did not demonstrate any café-au-lait macules or axillary or inguinal freckling but did reveal scattered melanocytic nevi. Additionally, no Lisch nodules or cutaneous neurofibromas were present. His mother is alive without a personal or significant family history of neoplasia. His father has a history of multifocal high-grade epithelial dysplasia of the oropharynx as well as resection of squamous cell carcinoma from the oral cavity. His sister died of glioblastoma at age 14, and his paternal grandfather had a history of cutaneous melanoma and died of brain cancer. Two paternal uncles are currently alive, one with a history of cutaneous melanoma and the other with a history of oral cancer. A pedigree of the paternal lineage is shown in Figure 1. 

**Figure 2.**
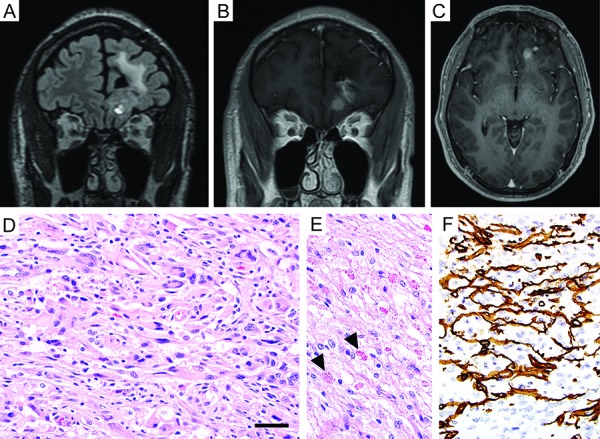
Imaging and histologic features of the pleomorphic xanthoastrocytoma resected from the left frontal lobe. A, B, C: Surveillance MR imaging 8 years after initial resection of a low-grade astrocytoma from the left frontal lobe demonstrated interval development of a new 7-mm focus of nodular enhancement adjacent to the prior resection cavity within the left medial orbital gyrus. Coronal T2/FLAIR-weighted image (A), coronal T1-weighted post-gadolinium image (B), and axial T1-weighted post-gadolinium image (C). D, E: Hematoxylin and eosin stained sections showing an astrocytic neoplasm with marked nuclear pleomorphism, numerous eosinophilic granular bodies (arrowheads), and occasional xanthomatous tumor cells with foamy cytoplasm. F: Immunostain for type IV collagen demonstrating abundant intercellular collagen deposition amongst the neoplastic astrocytes. Scale bar, 40 μm.

**Figure 3.**
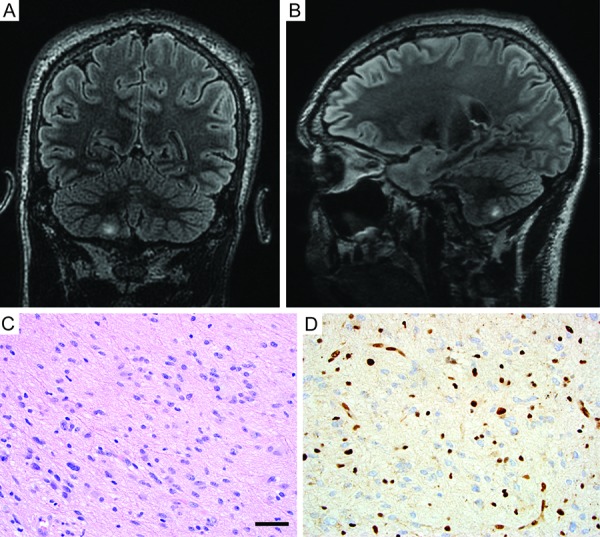
Imaging and histologic features of the diffuse astrocytoma resected from the right cerebellum. A, B: T2/FLAIR-weighted MR images demonstrating a hyperintense mass lesion in the right cerebellar hemisphere (coronal, A; sagittal, B). C: Hematoxylin and eosin stained section showing a diffuse glial neoplasm composed of neoplastic fibrillary astrocytes with elongate and irregular, hyperchromatic nuclei infiltrating through the cerebellar subcortical white matter. D: Immunohistochemistry for ATRX protein demonstrating absence of staining in the tumor cells with intact staining in entrapped non-neoplastic neurons and endothelial cells, consistent with somatic ATRX loss. Scale bar, 40 µm.

**Figure 4.**
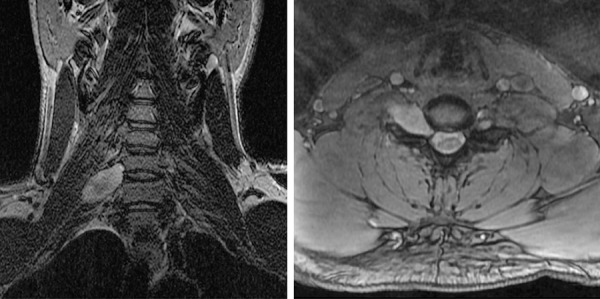
MR imaging of the cervical spine demonstrating an intradural, extramedullary mass centered in the right C6-C7 neural foramen and extending along the C7 nerve root (coronal T2-weighted, left; axial T1-weighted post-gadolinium, right).

After resection, no additional adjuvant therapy was administered, and he was monitored by regular MR imaging of the brain over the next several years. Aside from suffering from occasional seizures associated with poor compliance to anticonvulsant medications, the patient remained otherwise neurologically intact. A surveillance scan 8 years after his initial surgery, now at 31 years of age, revealed a new area of nodular enhancement with associated hemorrhage adjacent to the prior resection cavity in the left frontal lobe (Figure 2A, B, C). Additionally, a new T2- and fluid-attenuated inversion recovery (FLAIR) hyperintense mass lesion in the right cerebellar hemisphere was seen that did not enhance after contrast administration (Figure 3A, B). The patient underwent a gross total resection of the enhancing nodule in the left frontal lobe. Pathology demonstrated a solid, non-infiltrative astrocytic neoplasm with marked nuclear pleomorphism, occasional xanthomatous tumor cells with foamy cytoplasm, and numerous eosinophilic granular bodies (Figure 2D, E). The mitotic index was low, and high-grade histologic features including necrosis and microvascular proliferation were not identified. An immunostain for type IV collagen revealed abundant intercellular collagen deposition (Figure 2F). Additional immunostains revealed that the tumor cells had intact/retained expression of ATRX protein and were negative for IDH1-R132H mutant protein. Ki67 labeling was seen in ~ 2% of tumor cells. A diagnosis of pleomorphic xanthoastrocytoma (PXA), WHO grade II, was rendered. Given this diagnosis and the potential for PXA to disseminate in the cerebrospinal fluid throughout the neuraxis, the right cerebellar lesion was considered worrisome for disseminated disease versus possibly representing a second primary tumor. To further evaluate the patient, MR imaging of the spinal cord was also performed, which demonstrated an expansile, contrast-enhancing mass within the right C6-7 neural foramen tracking along the C7 nerve root, consistent with a peripheral nerve sheath tumor (Figure 4). A resection of the right cerebellar lesion was performed 1 month following the left frontal craniotomy. Pathology demonstrated a diffuse astrocytoma composed of neoplastic fibrillary astrocytes with elongate and irregular, hyperchromatic nuclei infiltrating through the subcortical white matter and internal granular layer of the cerebellum (Figure 3C). The tumor cells demonstrated absence of ATRX immunostaining with intact staining in entrapped non-neoplastic neurons and endothelial cells, consistent with ATRX loss (Figure 3D). The tumor cells were negative for IDH1-R132H and histone H3-K27M mutant proteins by immunohistochemistry. Ki67 labeling was present in ~ 5% of tumor cells. A diagnosis of diffuse astrocytoma, WHO grade II, was rendered. Given the significant family history and the presence of multiple histologically-distinct brain tumors and a peripheral nerve sheath tumor, genomic testing was recommended. 

After informed consent was obtained, targeted next-generation sequencing was performed on genomic DNA isolated from a peripheral blood sample and tumor tissue from the left frontal PXA and right cerebellar diffuse astrocytoma. This sequencing was performed on the UCSF500 Cancer Gene Panel as previously described, which utilizes capture-based next-generation sequencing targeting the coding regions of 479 cancer-associated genes along with select introns from 47 of these genes as well as the promoter region of the *TERT* gene [18]. 

**Figure 5.**
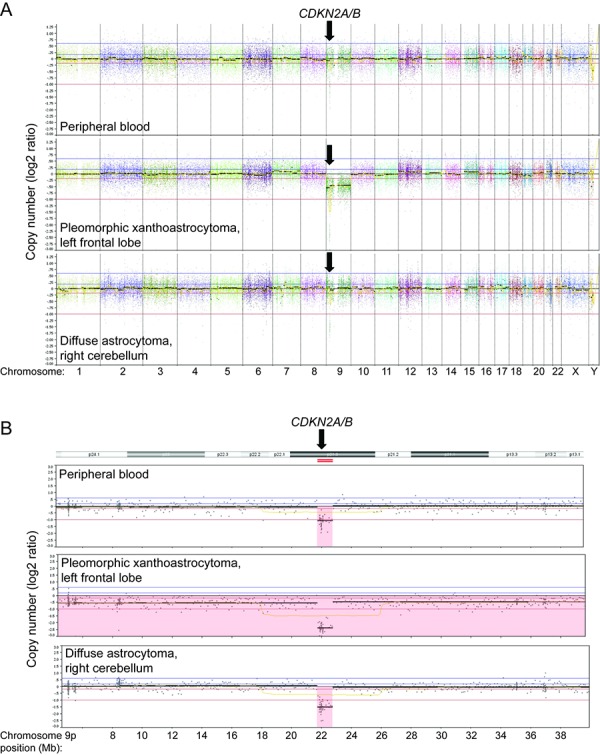
Genomic copy number analysis derived from targeted-capture next-generation sequencing data demonstrating heterozygous deletion on chromosome 9p21.3 containing the *CDKN2A* and *CDKN2B* tumor suppressor genes in the patient’s germline with somatic loss of chromosome 9p containing the remaining *CDKN2A/B* alleles in both astrocytic neoplasms. A: Genome-wide copy number plots of DNA extracted from peripheral blood (top), the left frontal pleomorphic xanthoastrocytoma (middle), and right cerebellar diffuse astrocytoma (bottom) demonstrating the focal deletion on chromosome 9p21, loss of chromosome 9 in the pleomorphic xanthoastrocytoma, and copy-neutral loss of heterozygosity of chromosome 9p in the diffuse astrocytoma, but no additional copy number alterations. B: Copy number plots for chromosome 9p highlighting identical boundaries of the focal 9p21.3 deletion within the peripheral blood and both tumors. The deletion is heterozygous within the peripheral blood and homozygous in both tumors due to somatic loss of the other copy of chromosome 9p.

This analysis identified a focal heterozygous deletion on chromosome 9p21.3 spanning approximate coordinates chr9: g.21,700,000 – 22,800,000 (human genome assembly GRCh38) within the peripheral blood sample (Figure 5). This deletion contains both the *CDKN2A* and *CDKN2B* tumor suppressor genes. The left frontal PXA demonstrated homozygous deletion of *CDKN2A/B* due to loss of the other copy of chromosome 9 containing the remaining intact *CDKN2A/B* alleles. This was accompanied by the p.V600E somatic hotspot mutation in *BRAF* (Table 1). Alterations involving *IDH1*, *IDH2*, *TP53*, and *ATRX* were not identified. The right cerebellar diffuse astrocytoma demonstrated homozygous deletion of *CDKN2A/B* due to copy-neutral loss of heterozygosity of chromosome 9p. This was accompanied by a hotspot activating missense mutation in *PTPN11*, two inactivating frameshift mutations in the *NF1* tumor suppressor gene, and an inactivating frameshift mutation in the *ATRX* tumor suppressor gene. No alterations involving *IDH1*, *IDH2*, or *TP53* were identified, nor was the *BRAF* p.V600E mutation found in the PXA. Apart from the copy number changes involving chromosome 9, no other chromosomal copy number aberrations were seen in either tumor. 

The presence of germline *CDKN2A/B* inactivation together with the presence of multiple anatomically, histologically, and genetically distinct astrocytic neoplasms, both with accompanying somatic loss of the remaining *CDKN2A/B* alleles, is diagnostic of familial melanoma-astrocytoma syndrome. This genetic diagnosis is further supported by the significant family history of both glioblastoma and melanoma in the paternal lineage, suggesting that this is likely an inherited rather than de novo germline alteration in this patient. The patient and his family have been referred to a medical geneticist with expertise in cancer risk evaluation; however, no genetic evaluation of any family members has yet been performed. Given his newly-identified tumor predisposition syndrome, the patient has initiated the recommended cancer screening, including annual dermatologic, ophthalmologic, and dental examinations to monitor for the development of cutaneous, ocular, and mucosal melanoma. He will continue to undergo regular surveillance imaging of the brain. Additionally, imaging of the chest, abdomen, and pelvis was performed to rule out the presence of visceral malignancy, which was unrevealing except for the presence of the paraspinal peripheral nerve sheath tumor that remains asymptomatic and is being radiographically monitored at present. 

## Discussion 

This is the first report, to our knowledge, to demonstrate multiple distinct histologic subtypes of astrocytoma in a patient with familial melanoma-astrocytoma syndrome. The histologic features of the astrocytomas that arise as part of this syndrome are not well described, and pleomorphic xanthoastrocytoma has not been previously described in association with familial melanoma-astrocytoma syndrome. We document the presence of two anatomically-distinct neoplasms, one with histologic features diagnostic of pleomorphic xanthoastrocytoma and one with histologic features diagnostic of diffuse astrocytoma. The pathologic findings in this patient thus expand the histologic diversity of astrocytic neoplasms that can be seen as part of this syndrome, which can range from pleomorphic xanthoastrocytoma to diffuse or anaplastic astrocytoma to glioblastoma. We are not aware of any reports describing tumors histologically resembling other glial neoplasms, including pilocytic astrocytoma, subependymal giant cell astrocytoma, ganglioglioma, or oligodendroglioma in patients with this syndrome. 

Additionally, we describe for the first time the cooperating somatic alterations that drive gliomagenesis in those astrocytomas arising as part of familial melanoma-astrocytoma syndrome in addition to germline inactivation of *CDKN2A*. In the pleomorphic xanthoastrocytoma, we show that the germline *CDKN2A/B* deletion was accompanied by somatic loss of the remaining *CDKN2A/B* alleles as well as the activating p.V600E hotspot mutation in *BRAF*. Together, *CDKN2A* deletion and *BRAF* p.V600E are the two characteristic somatic alterations seen in the majority of sporadic pleomorphic xanthoastrocytomas [19]. In the diffuse astrocytoma, we show that the germline *CDKN2A/B* deletion was accompanied by somatic loss of the remaining *CDKN2A/B* alleles as well as an activating hotspot mutation in *PTPN11* and inactivating frameshift mutations in the *ATRX* and *NF1* tumor suppressor genes. *ATRX* mutations are present in greater than 90% of diffuse lower-grade astrocytomas and are frequently accompanied by *TP53* and *IDH1* or *IDH2* mutations [20]. The diffuse astrocytoma in this patient lacked alterations in *TP53*, *IDH1*, and *IDH2*. Instead, the additional cooperating mutations involved *PTPN11* and *NF1*. Amongst sporadic gliomas, *PTPN11* mutations are rare and most commonly found in pilocytic astrocytomas, whereas *NF1* mutations are very common in primary glioblastomas but not typically seen in diffuse lower-grade astrocytomas [19, 20, 21]. Somatic *CDKN2A* deletions are common in both diffuse lower-grade astrocytomas and primary glioblastomas [20, 21]. Thus, while the genetic alterations seen in this patient’s pleomorphic xanthoastrocytoma are typical for this tumor type, the genetic alterations seen in the diffuse astrocytoma are somewhat unusual and make prognostic classification based on the molecular profile challenging [22]. 

Regarding management, optimal treatment strategies for patients with familial melanoma-astrocytoma syndrome are unknown. While specific recommendations regarding disease surveillance have not been established given the rarity of this syndrome, these patients require regular brain imaging to monitor for astrocytoma development; dermatologic and ophthalmologic evaluation to monitor for cutaneous and ocular melanoma development; dental exam to monitor for oropharyngeal dysplasia and mucosal melanoma; and body imaging to monitor for development of visceral malignancies, in particular pancreatic carcinoma. Additionally, genetic counseling for the patient and their families is essential. 

In summary, we present a rare case of a patient with familial melanoma-astrocytoma syndrome who was found to have synchronous development of two anatomically, histologically, and genetically distinct astrocytomas as well as a presumed peripheral nerve sheath tumor. Our report highlights the histologic spectrum of astrocytic neoplasms that can be seen as part of this syndrome, as well as defining the cooperating genetic alterations that drive these astrocytomas. 

## Acknowledgment 

D.A.S. is supported by the NIH Director’s Early Independence Award (DP5 OD021403). 

## Conflict of interest 

The authors have no conflicts of interest related to this case report to disclose. 

**Table 1.** Somatic alterations identified in the left frontal pleomorphic xanthoastrocytoma and right cerebellar diffuse astrocytoma.

Pleomorphic xanthoastrocytoma, left frontal lobe		
Variant	Reference transcript	Classification
*CDKN2A/B* homozygous deletion	N/A	Pathogenic
*BRAF* p.V600E	NM_004333	Pathogenic
*CDH1* p.V55G	NM_004360	VUS
*SPTA1* p.T1953I	NM_003126	VUS
Diffuse astrocytoma, right cerebellum		
Variant	Reference transcript	Classification
*CDKN2A/B* homozygous deletion	N/A	Pathogenic
*ATRX* p.G1152fs	NM_000489	Pathogenic
*NF1* p.L1246fs	NM_001042492	Pathogenic
*NF1* p.W1685fs	NM_001042492	Pathogenic
*PTPN11* p.T73I	NM_002834	Pathogenic

fs = frameshift; N/A = not applicable; VUS = variant of unknown significance.
